# Prävention von Pflegebedürftigkeit: Analysen von Begutachtungsdaten des Medizinischen Dienstes

**DOI:** 10.1007/s00103-023-03685-5

**Published:** 2023-03-21

**Authors:** Stefan Blüher, Ralph Schilling, Thomas Stein, Paul Gellert

**Affiliations:** 1 Institut für Medizinische Soziologie und Rehabilitationswissenschaft, Charité – Universitätsmedizin Berlin, Charitéplatz 1, 10117 Berlin, Deutschland; 2 Institut für Sozialmedizin, Epidemiologie und Gesundheitsökonomie, Charité – Universitätsmedizin Berlin, Berlin, Deutschland

**Keywords:** Determinanten von Pflegebedürftigkeit, Präventive Potenziale, Begutachtungsdaten, Medizinischer Dienst, Determinants of the need for long-term care, Preventive potential, Assessment data of the Medical Service

## Abstract

**Hintergrund:**

Die Bedeutung der Prävention von Pflegebedürftigkeit wächst mit der Zunahme von pflegebedürftigen Menschen. Für Deutschland gibt es bislang nur unzureichende Daten darüber, welche Faktoren mit der Entstehung einer Pflegebedürftigkeit assoziiert sind. Die vorliegende Studie untersucht die Wechselwirkungen zwischen soziodemografischen und gesundheitsbezogenen Determinanten von Pflegebedürftigkeit, um daraus präventive Anknüpfungspunkte abzuleiten.

**Methoden:**

Analysiert wurden die Begutachtungsdaten des Medizinischen Dienstes (MD) Berlin-Brandenburg zur Feststellung einer Pflegebedürftigkeit nach SGB XI für die Zeiträume 2017 und 2018/2019 mit Fokus auf die Antragstellenden, die über den betrachteten Zeitraum hinweg ohne Pflegegradeinstufung blieben (6037 von insg. 72.680 Antragstellenden des Jahres 2017). Soziale Faktoren wie Haushaltszusammensetzungen, Unterstützungspotenziale und Partnerschaftsstatus wurden über Text-Mining-Verfahren extrahiert und die Daten mittels deskriptiver und multivariabler statistischer Verfahren ausgewertet.

**Ergebnisse:**

Eine erhöhte Chance, ohne Pflegegradeinstufung zu bleiben, hatten jüngere Antragstellende sowie Personen ohne Partnerschaft. Im Zeitraum 2018/2019 ebenfalls assoziiert mit einer erhöhten Chance, ohne Pflegegrad zu bleiben, waren eine Verbesserung der Gesundheit, seit 2017 ohne soziale Unterstützung zu sein, muskuloskelettale Erkrankungen sowie chronisch ischämische Herzkrankheiten. Geringere Chancen, ohne Pflegegradeinstufung zu bleiben, hatten hingegen Antragstellende mit demenziellen und sonstigen psychischen Erkrankungen.

**Diskussion:**

Die erstmalige Untersuchung der MD-Begutachtungsdaten unter einer präventiven Perspektive zeigt, dass soziodemografische und gesundheitsbezogene Determinanten in Wechselwirkung betrachtet werden müssen, um zusätzliche Präventionspotenziale auszumachen

## Abstract (en)

**Background:**

The importance of preventing the need for care increases with the growing number of people in need of care. For Germany, there is currently insufficient data on which factors are associated with the need for long-term care. In order to derive a preventive approach, this study examines the interactions between sociodemographic and health-related factors that determine the need for long-term care.

**Methods:**

We analyzed the assessment data of the Medical Service Berlin-Brandenburg, which determines a need for care according to SGB XI for the 2017 and 2018/19 periods. We focused on the applicants who remained without a care grade classification over the period under consideration (6037 out of a total of 72,680 applicants in 2017). Social factors such as household composition, support potential, and partnership status were extracted using text-mining methods, and the data was evaluated using descriptive and multivariable statistical methods.

**Results:**

Younger applicants and people without a partner had an increased chance of not being diagnosed with a need for long-term care. Also associated with an increased chance of remaining without a degree of care in 2018/19 were an improvement in health, having been without social support since 2017, musculoskeletal disorders, and chronic ischemic heart disease. On the other hand, applicants with dementia and other mental illnesses had fewer chances of remaining without a care level classification.

**Discussion:**

The first investigation of the Medical Service assessment data from a preventive perspective shows that the interaction of sociodemographic and health-related determinants must be considered in order to identify additional preventive potential.

## Hintergrund

Die zukünftig deutlich steigende Nachfrage nach Pflegeleistungen und absehbare Versorgungslücken stellen große Herausforderungen an unser Gesundheitssystem und die Gesellschaft insgesamt. Die fortschreitende Bevölkerungsalterung zeigt sich heute in der absolut und relativ gestiegenen Zahl älterer Menschen (über 60-Jährige) und innerhalb dieser Population insbesondere auch in der deutlichen Zunahme der Zahl Hochaltriger (über 85-Jährige) und Langlebiger (über 100-Jährige; [1]). Die gestiegene Lebenserwartung beeinflusst das Risiko des Eintritts von Pflegebedürftigkeit im Lebensverlauf. Einerseits verlängert sich die Lebenszeit, die in (relativ) gutem Gesundheitszustand verbracht wird. Damit verschiebt sich auch das durchschnittliche Alter, in dem Pflegebedarf entsteht, teilweise weiter in die Hochaltrigkeit hinein. Diese Veränderungen führen jedoch nicht zu einer zunehmenden Kompression des Zeitraums von Hilfe- und Pflegebedarf vor dem Lebensende, da demgegenüber die Zuwächse an durchschnittlicher und fernerer Lebenserwartung höher sind [1]. Dadurch – so ist seit einigen Jahren zu beobachten – verlängern sich auch die Abschnitte des Lebens mit einem Pflegebedarf. Die gewonnene Lebenszeit bringt sowohl einen Zugewinn an gesunden Lebensabschnitten als auch eine Verlängerung von Lebensabschnitten mit Pflegebedarf [2].

Zudem weist die Entwicklung in Deutschland mit der Generation der Babyboomer eine demografische Besonderheit auf, die die Szenarien zur Pflegebedürftigkeit zusätzlich beeinflusst. Die seit dem Zweiten Weltkrieg historisch stärksten Geburtsjahrgänge der Kohorten der 1955er- bis 1965er-Jahre werden die gesundheits- und sozialpolitischen Herausforderungen der kommenden Jahrzehnte maßgeblich prägen. So werden die Babyboomer um das Jahr 2045 zwischen 80 und 90 Jahre alt sein und somit in den Altersgruppen mit höherem Pflegerisiko liegen. Entsprechend ist eine deutliche Zunahme der Nachfrage nach pflegerischen Leistungen zu erwarten. Absehbare Versorgungslücken zwischen Angeboten pflegerischer Versorgung und steigender Nachfrage werden derzeit vor allem hinsichtlich eines erforderlichen Ausbaus der ambulanten und (teil-)stationären pflegerischen Versorgungsstruktur und des sich gleichzeitig verschärfenden Fachkräftemangels diskutiert. Die politische Gestaltung fokussiert damit deutlich auf die Weiterentwicklung und den Ausbau der Strukturen auf der *Angebotsseite *pflegerischer Versorgung. So sehr dies notwendig ist, so sehr verweisen Befunde auf die Begrenztheit dieser Ansätze: Der Sachverständigenrat berechnete schon 2012 in seinem Gutachten [3] einen Fachkräftemangel in der Pflege für 2025, den er mit ca. 200.000 bezifferte – und dies angesichts eines massiven Rückgangs der Erwerbsbevölkerung in Deutschland insgesamt [1].

Vor dem Hintergrund dieser und weiterer personeller, finanzieller und struktureller Limitationen auf der Angebotsseite pflegerischer Versorgung darf die Gestaltbarkeit der Nachfrageseite nicht aus dem Blick geraten. Vielmehr sind – gerade aus einer Public-Health-Perspektive – verstärkte Bemühungen notwendig, um die Nachfrage nach Pflegeleistungen abzusenken. Konkret bedeutet dies, gesundheitsförderliche, präventive und rehabilitative Potenziale vor der Entstehung einer Pflegebedürftigkeit zu identifizieren und zu stärken, um Pflegebedarf zu vermeiden, zu verzögern oder Verläufe abzumildern. So zeigen beispielsweise Hajek et al. (2017) in einer bevölkerungsbasierten prospektiven Kohortenstudie bedeutsame Prädiktoren von Pflegebedürftigkeit im Längsschnitt: Demenzielle Erkrankungen, Beeinträchtigungen in der Mobilität und ein höheres Alter sind dabei maßgebliche Faktoren, die die Wahrscheinlichkeit einer Pflegebedürftigkeit erhöhen [4]. Soziale Faktoren wie der Familienstand, die Wohnsituation, aber auch sensorische und mentale Faktoren, wie Sehbeeinträchtigungen und Depressionen hatten einen geringeren Einfluss; Schwerhörigkeit war nicht mit einer erhöhten Pflegbedürftigkeit assoziiert. Gellert et al. (2018) zeigten in einer prospektiven Kohortenstudie und longitudinalen Analysen basierend auf Routinedaten der gesetzlichen Kranken- und Pflegeversicherung, dass Alter, Krankenhauseinweisungen und demenzielle Erkrankungen positiv, während muskuloskelettale Erkrankungen negativ mit der Langzeitpflege assoziiert waren [5]. Die Assoziation mit Demenz war bei 100-Jährigen signifikant schwächer ausgeprägt als bei den jüngeren Vergleichskohorten hochaltriger Menschen. Blüher et al. (2017) konstatieren neben Alter, Krankheitsentwicklungen, sozialen Determinanten, Mobilitätseinschränkungen, Sturzereignissen und Inkontinenz auch psychische Erkrankungen, Aspekte des Wohlbefindens sowie subjektive Gesundheitseinschätzungen als Determinanten von Pflegebedürftigkeit im Alter; mögliche Wechselwirkungen dieser Determinanten sind hingegen noch unzureichend untersucht [6]. Weiterhin haben die genannten Datenquellen aus den Kohortenstudien Schwächen (z. B. selektiver Ausfall von Teilnehmenden, fehlende Werte bei Befragungskohorten, keine sozialen Informationen bei Routinedaten der gesetzlichen Kranken- und Pflegeversicherung), die nach neuen Ansätzen verlangen. Bedeutsame Faktoren für die Entstehung einer Pflegebedürftigkeit zu kennen und auch mögliche Wechselwirkungen zwischen physischen, psychischen und sozialen Komponenten, die einen Pflegebedarf determinieren, zu analysieren, sollte Gegenstand künftiger Forschung sein.

Ziel der vorliegenden Studie war es, die soziodemografischen und gesundheitsbezogenen Determinanten mit Blick auf die Verhinderung einer Pflegebedürftigkeit zu untersuchen. Im Vordergrund standen dabei soziale Determinanten, wie etwa der Partnerschaftsstatus. Durch die erstmalige Verwendung der Datensätze aus Begutachtungen des Medizinischen Dienstes (MD) wurden auch Möglichkeiten und Grenzen dieses Datentyps für die Präventions‑, Public-Health- und Versorgungsforschung ausgelotet. Es wurde dezidiert die Gruppe von Antragstellenden untersucht, die über den betrachteten Zeitraum hinweg ohne Pflegegradeinstufung blieben.

## Methoden

Ein vom Spitzenverband der Gesetzlichen Krankenversicherung gefördertes und vom Institut für Medizinische Soziologie und Rehabilitationswissenschaft der Charité – Universitätsmedizin Berlin durchgeführtes Forschungsprojekt „Gesundheitsverläufe im Alter: Wege in die Pflegebedürftigkeit“ (März 2018 bis September 2022) analysierte umfangreiche Datensätze aus Begutachtungen des MD Berlin-Brandenburg zur Feststellung einer Pflegebedürftigkeit nach dem Elften Buch Sozialgesetzbuch SGB XI [7, 8]. Das vom MD verwendete Begutachtungsinstrument enthält neben soziodemografischen Daten auch Angaben über die pflegerelevante Vorgeschichte und die aktuelle Versorgungssituation sowie teilweise Beschreibungen zur Wohnsituation, Haushaltszusammensetzungen und Informationen zu Unterstützungspotenzialen, etwa zu Partnerschaften oder anderen Angehörigen. Diese Informationen sowie die im Begutachtungsinstrument angegebenen pflegebegründenden Erst- und Zweitdiagnosen führen zur Einschätzung der Fähigkeit der antragstellenden Person, Aufgaben und Anforderungen des Alltags selbständig durchzuführen. Diese Einschätzung erfolgt dabei differenziert nach 6 definierten und unterschiedlich gewichteten Modulen:

1. Mobilität (Gewichtung 10 %),
2. kognitive und kommunikative Fähigkeiten,
3. Verhaltensweisen und psychische Problemlagen (15 %, höchster Punktwert aus Modul 2 und 3 wird verwendet),
4. Selbstversorgung (40 %),
5. Bewältigung von und selbständiger Umgang mit krankheits- und therapiebedingten Anforderungen und Belastungen (20 %) sowie
6. Gestaltung des Alltagslebens und sozialer Kontakte (15 %; [9]).

Anhand der Bewertung des Grades an Selbständigkeit der antragstellenden Person wird letztlich die Einstufungsempfehlung in einen Pflegegrad vorgenommen, respektive keine Pflegebedürftigkeit festgestellt.

Im Rahmen der Studie konnten 2 Datensätze des MD Berlin-Brandenburg ausgewertet werden:

1. Erstbegutachtungen des Jahres 2017 aus den Bundesländern Berlin und Brandenburg. Der zur Verfügung gestellte Datensatz umfasste insgesamt 72.680 Antragstellende im Alter von 50 bis 99 Jahren.

Ein analyseleitender Grundgedanke der Studie bestand darin, dass im Begutachtungsverfahren die Gruppe von Antragstellenden von besonderem Interesse ist, die nach Prüfung *keinen* Pflegegrad zuerkannt bekommt. Aus der Charakterisierung dieser Gruppe lassen sich in Kontrastierung zur Gruppe mit Feststellung einer Pflegebedürftigkeit – so die Überlegung – spezifische Ressourcen und Risiken beschreiben und präventive oder rehabilitative Anknüpfungen identifizieren, die möglicherweise den Eintritt von Pflegebedarf vermeiden oder verzögern.

2. Vor diesem Hintergrund konnte ein zweiter, zusammengefasster Datensatz des MD der Jahre 2018/2019 mit insgesamt 11.423 Begutachtungen von 7657 Antragstellenden einbezogen werden. Hiervon ausgeschlossen wurden 1259 Fälle, bei denen im Jahr 2017 ein Pflegegrad größer 0 vorlag. Des Weiteren wurden 361 Fälle ausgeschlossen, die nicht in eigener Häuslichkeit wohnten. Somit verblieben 6037 in der eigenen Häuslichkeit wohnende Personen, bei denen im Jahr 2017 keine Pflegebedürftigkeit festgestellt worden war und die im Zeitraum 2018/2019 (mindestens) einen weiteren Antrag auf Leistungen aus der Pflegeversicherung gestellt hatten und infolgedessen erneut begutachtet wurden. Es bestand somit die Möglichkeit, diese im Fokus der Analyse stehende Gruppe der Antragstellenden ohne Pflegegrad im Zeitverlauf von 2017 bis Ende 2019 zu verfolgen. Die Analysen basieren auf dem Ergebnis der letzten Begutachtung im Zeitraum 2018/2019.

### Statistische Analyse

Mit Blick auf die Kontrastierung der Antragstellenden mit und ohne Pflegegrad im Zeitraum 2018/2019 wurde für die vorliegenden Analysen mittels binärlogistischer Regression die Wahrscheinlichkeit berechnet, zur Gruppe mit oder ohne Feststellung einer Pflegebedürftigkeit zu gehören. Die Ausprägung „mit Feststellung einer Pflegebedürftigkeit“ ist dabei als Referenzkategorie (Ref.) festgelegt, die Ergebnisse werden somit für die Gruppe der „Antragstellenden ohne Pflegegradempfehlung“ ausgewiesen. Als Prädiktoren sind die Merkmale Alter und Geschlecht sowie Partnerschaftsstatus und häufigste Erstdiagnosen im Zeitraum 2018/2019 (nach ICD-10-Codierung) in die Modellierung einbezogen. Um Veränderungen in der Verfügbarkeit sozialer Unterstützung sowie Veränderungen im Krankheitsgeschehen (gemessen an der Anzahl der Diagnosen) über den Zeitverlauf von 2017 bis 2018/2019 abbilden zu können, wurden die entsprechenden Informationen aus beiden zur Verfügung stehenden Datensätzen in Variablen aggregiert und ebenfalls in die Modellierung einbezogen.

Die Informationen zu sozialen Bezügen (Haushaltszusammensetzungen, Unterstützungspotenziale, Partnerschaftsstatus) lagen nicht in standardisierter Form vor, sondern mussten zunächst durch Anwendung eines Text-Mining-Verfahrens mithilfe einer Verschlagwortung von Schlüsselbegriffen aus den Freitextangaben extrahiert und einer statistischen Analyse zugänglich gemacht werden. Dazu werden thematisch relevante Freitextangaben in den Datensätzen der Antragstellenden selektiert und in Zeichenketten (Strings) umgewandelt. Anschließend wird jeder dieser Strings exakt nach relevanten synonymen Schlagwörtern durchsucht [10]. Dementsprechend beziehen sich die Analysen ausschließlich auf Unterstützungspotenziale wie Partnerschaften und soziale Netzwerke, sofern entsprechende Hinweise darauf in den Freitexten enthalten waren.

Die Analysen wurden mit der Statistiksoftware IBM SPSS Version 26 durchgeführt. Im Rahmen der deskriptiven Analysen sind absolute und relative Häufigkeiten für kategoriale Variablen ausgewiesen. Für die multivariablen Analysen wurden neben adjustierten Odds Ratios (OR) außerdem 95 %-Konfidenzintervalle (KI) und *p*-Werte berechnet. Die Analysen sind zweiseitig zum Signifikanzniveau von α = 5 % durchgeführt, fehlende Werte nicht ersetzt. Die Ergebnisse werden explorativ interpretiert.

## Ergebnisse

### Charakteristika von Antragstellenden im Zeitverlauf 2017 bis 2018/2019

Aus den Begutachtungen des Jahres 2017 gingen von den insgesamt 72.680 Antragstellenden 15.108 Personen ohne Pflegegradeinstufung hervor; von diesen sind 9138 (60,5 %) weiblich und 5970 (39,5 %) männlich. Insgesamt 6133 (40,6 %) sind 50 bis unter 75 Jahre alt, 8975 (59,4 %) sind 75 Jahre alt und älter, davon 710 (4,7 %) im Alter von 90 Jahren und mehr. Zu Hause alleinlebend waren 10.020 (67,7 %), während 4786 (32,3 %) Antragstellende berichteten, in eigener Häuslichkeit mit mindestens einer weiteren Person zusammenzuleben. Mit Blick auf die Diagnosen fällt auf, dass die demenziellen Erkrankungen in der Gruppe ohne Pflegegrad praktisch keine Rolle spielen (0,9 %). Vielmehr dominieren hier muskuloskelettale Erkrankungen (Rückenschmerz, Polyarthrose, sonstige Arthrosen) sowie chronisch obstruktive Lungenerkrankung (COPD) und Herzinsuffizienz. Für die Gruppe derjenigen mit Pflegegrad hingegen stellen die demenziellen Erkrankungen eine überproportional bedeutsame Diagnose dar (9,0 %).

Die Daten aus den Begutachtungen im Zeitraum 2018/2019 boten die Möglichkeit, insgesamt 6037 Antragstellende ohne Pflegegrad aus dem Jahr 2017 weiterzuverfolgen, die 2018/2019 einen erneuten Antrag auf die Anerkennung einer Pflegebedürftigkeit gestellt haben. Die Geschlechter- und Altersverteilungen beider Datensätze weisen dabei keine nennenswerten Unterschiede auf. Es zeigt sich außerdem, dass 1104 (18,3 %) der Antragstellenden bis Ende 2019 trotz erneuter Begutachtung weiterhin ohne Pflegegrad blieben, während 2305 (38,2 %) in Pflegegrad 1, 2201 (36,5 %) in Pflegegrad 2, 383 (6,3 %) in Pflegegrad 3, 38 (0,6 %) in Pflegegrad 4 und 6 Personen (0,1 %) in Pflegegrad 5 eingestuft wurden. Die Tab. 1 und Tab. 2 weisen darüber hinaus die Verteilungen für gesundheitsbezogene und soziale Faktoren für diese Antragstellenden mit und ohne Pflegegradeinstufung im Zeitraum 2018/2019 aus. Die oben beschriebenen Merkmale von Antragstellenden ohne Pflegegrad aus dem Jahr 2017 zeigen sich demnach auch Ende 2019 als bedeutsam für ein geringeres Risiko im Hinblick auf die Anerkennung von Pflegebedürftigkeit: Es sind wiederum Jüngere (unter 75-Jährige) und Personen ohne kognitive und psychische Einschränkungen, die mit höherer Wahrscheinlichkeit ohne Pflegegrad bleiben. Auch leben Antragstellende ohne Pflegegradeinstufung 2018/2019 häufiger ohne Hilfe allein im eigenen Haushalt bzw. berichten seltener von einer Partnerschaft.

**Table 1:** 

Pflegegrad	Ohne	Mit (1–5)	1	2	3	4 + 5	Gesamt (ohne + mit)
*Geschlecht*							
Weiblich, *n* (%)	*668 (60,5)*	*3171 (64,3)*	1526 (66,2)	1390 (63,2)	229 (59,8)	25 (56,8)	(63,6)
Männlich, *n* (%)	*436 (39,5)*	*1762 (35,7)*	779 (33,8)	811 (36,8)	154 (40,2)	19 (43,2)	2199 (36,4)
*Bundesland*							
Berlin, *n* (%)	*732 (66,3)*	*3011 (61,0)*	1447 (62,8)	1327 (60,3)	216 (56,4)	21 (47,7)	3743 (62,0)
Brandenburg, *n* (%)	*372 (33,7)*	*1922 (39,0)*	858 (37,2)	874 (39,7)	167 (43,6)	23 (52,3)	2294 (38,0)
*Altersgruppe*							
50–64, *n* (%)	*281 (25,5)*	*581 (11,8)*	321 (13,9)	220 (10,0)	34 (8,9)	6 (13,6)	862 (14,3)
65–74, *n* (%)	*267 (24,2)*	*737 (14,9)*	368 (16,0)	308 (14,0)	56 (14,6)	5 (11,4)	1004 (16,6)
75–89, *n* (%)	*502 (45,5)*	*3116 (63,2)*	1444 (62,6)	1402 (63,7)	244 (63,7)	26 (59,1)	3618 (59,9)
90+, *n* (%)	*54 (4,9)*	*499 (10,1)*	172 (7,5)	271 (12,3)	49 (12,8)	7 (15,9)	553 (9,2)
*Soziale Unterstützung*							
Vorhanden, *n* (%)	*773 (70,0)*	*3766 (76,3)*	1702 (73,8)	1693 (76,9)	328 (85,6)	43 (97,7)	4539 (75,2)
Nicht vorhanden, *n* (%)	*331 (30,0)*	*1167 (23,7)*	603 (26,2)	508 (23,1)	55 (14,4)	1 (2,3)	1498 (24,8)
*Haushaltszusammensetzung*							
Alleinlebend ohne Hilfe, *n* (%)	*305 (27,6)*	*1071 (21,7)*	564 (24,5)	464 (21,1)	42 (11,0)	1 (2,3)	1376 (22,8)
Alleinlebend mit Hilfe, *n* (%)	*517 (46,8)*	*2387 (48,4)*	1138 (49,4)	1058 (48,1)	176 (46,0)	15 (34,1)	2904 (48,1)
Mit weiterer Person zusammenlebend, *n* (%)	*282 (25,5)*	*1475 (29,9)*	603 (26,2)	679 (30,8)	165 (43,1)	28 (63,6)	1757 (29,1)
*Partnerschaft*							
Mit Partner*in, *n* (%)	*266 (24,1)*	*1506 (30,5)*	626 (27,2)	703 (31,9)	156 (40,7)	21 (47,4)	1772 (29,4)
Ohne Partner*in, *n* (%)	*838 (75,9)*	*3427 (69,5)*	1679 (72,8)	1498 (68,1)	227 (59,3)	23 (52,6)	4265 (70,6)
*Häufigste Diagnosen*							
Demenz, *n* (%)	*2 (0,2)*	*255 (5,2)*	57 (2,5)	122 (5,5)	70 (18,3)	6 (13,6)	257 (4,3)
Polyarthrose, *n* (%)	*57 (5,2)*	*375 (7,6)*	176 (7,6)	172 (7,8)	25 (6,5)	2 (4,5)	432 (7,2)
Chronisch obstruktive Lungenerkrankung (COPD), *n* (%)	*88 (8,0)*	*341 (6,9)*	178 (7,7)	147 (6,7)	16 (4,2)	–	429 (7,1)
Herzinsuffizienz, *n* (%)	*54 (4,9)*	*282 (5,7)*	118 (5,1)	146 (6,6)	18 (4,7)	–	336 (5,6)
Rückenschmerzen, *n* (%)	*84 (7,6)*	*188 (3,8)*	105 (4,6)	79 (3,6)	2 (0,5)	2 (4,5)	272 (4,5)
Sonstige Arthrose, *n* (%)	*33 (3,0)*	*207 (4,2)*	101 (4,4)	96 (4,4)	9 (2,4)	1 (2,3)	240 (4,0)
Hirninfarkt, *n* (%)	*32 (2,9)*	*149 (3,0)*	64 (2,8)	66 (3,0)	16 (4,2)	3 (6,8)	181 (3,0)
Sonstige psychische Störungen, *n* (%)	*9 (0,8)*	*172 (3,5)*	57 (2,5)	79 (3,6)	30 (7,8)	6 (13,6)	181 (3,0)
Gonarthrose, *n* (%)	*48 (4,3)*	*120 (2,4)*	77 (3,3)	41 (1,9)	2 (0,5)	–	168 (2,8)
Chronische ischämische Herzkrankheit, *n* (%)	*36 (3,3)*	*105 (2,1)*	55 (2,4)	45 (2,0)	4 (1,0)	1 (2,3)	141 (2,3)
Andere, *n* (%)	*661 (59,9)*	*2739 (55,5)*	1317 (57,1)	1208 (54,9)	191 (49,9)	23 (52,3)	3400 (56,3)

**Table 2:** 

Pflegegrad	Ohne	Mit (1–5)	1	2	3	4 + 5	Gesamt (ohne + mit)
*Veränderung soz. Unterstützung in 2019*^*a*^							
Seit 2017 ohne soziale Unterstützung, *n* (%)	*211 (19,1)*	*595 (12,1)*	314 (13,7)	259 (11,8)	21 (5,5)	1 (2,3)	806 (13,4)
Seit 2017 mit sozialer Unterstützung, *n* (%)	*651 (59,0)*	*3241 (65,9)*	1437 (62,7)	1476 (67,2)	290 (75,9)	38 (86,4)	3892 (64,7)
In 2018/2019 mit sozialer Unterstützung, *n* (%)	*94 (8,5)*	*470 (9,6)*	244 (10,6)	205 (9,3)	21 (5,5)	–	564 (9,4)
In 2018/2019 ohne soziale Unterstützung, *n* (%)	*147 (13,3)*	*609 (12,4)*	298 (13,0)	256 (11,7)	50 (13,1)	5 (11,4)	756 (12,5)
*Veränderung der Anzahl von Diagnosen in 2019*^*b*^							
Seit 2017 eine Diagnose, *n* (%)	*–*	*–*	–	–	–	–	–
Seit 2017 mind. 2 Diagnosen, *n* (%)	*851 (77,1)*	*4388 (89,0)*	1997 (86,6)	1996 (90,7)	353 (92,2)	42 (95,4)	5239 (86,8)
In 2019 Reduktion zu einer Diagnose, *n* (%)	*253 (22,9)*	*545 (11,0)*	308 (13,4)	205 (8,3)	30 (7,8)	2 (4,6)	798 (13,2)
In 2019 Veränderung zu mehreren Diagnosen, *n* (%)	*–*	*–*	–	–	–	–	–

^a^Kombinationsvariable aus Haushaltszusammensetzung und sozialer Unterstützung

^b^Da 2 der Kategorien keine Fälle aufwiesen, wurde die Variable für die weiterführenden Analysen dichotomisiert

### Ergebnisse der multivariablen Analyse

Auch bei statistischer Kontrolle im Rahmen der multivariablen Analyse bleiben die Befunde zum Zusammenhang von gesundheitsbezogenen und sozialen Einflussfaktoren auf die Nichtfeststellung einer Pflegebedürftigkeit stabil. Tab. 3 verdeutlicht, dass insbesondere kognitive Einbußen ein zentrales Risiko für den Verlust von Selbständigkeit darstellen. Gegenüber Antragstellenden mit der Diagnose COPD (Ref.) haben Demenzerkrankte eine Chance von 4 % (OR 0,04; 95 %-KI 0,01–0,18), ohne Pflegebedürftigkeit zu bleiben; bei Antragstellenden mit einer Diagnose aus dem Bereich sonstiger psychischer Erkrankungen beträgt diese Chance etwa ein Drittel (OR 0,31; 95 %-KI 0,15–0,63). Ebenso deutlich zeigt sich, dass die Einstufung in einen Pflegegrad mit zunehmendem Alter wahrscheinlicher wird. Die Ergebnisse zeigen außerdem, dass Antragstellende ohne Partnerschaft mit höherer Wahrscheinlichkeit ohne Pflegegrad bleiben (OR 1,28; 95 %-KI 1,07–1,52) und häufiger berichten, im Zeitverlauf 2017 bis 2019 unverändert über kein Unterstützungspotenzial zu verfügen (OR 1,36; 95 %-KI 1,06–1,75). Veränderungen im Krankheitsgeschehen über den Zeitverlauf von 2017 bis 2018/2019 sind ebenfalls mit der Feststellung einer Pflegebedürftigkeit assoziiert. Demnach ist die Chance, ohne Pflegebedürftigkeit zu bleiben, bei Antragstellenden, die 2019 nur noch eine Diagnose aufweisen, im Vergleich zur Referenzgruppe um das Zweieinhalbfache erhöht.

**Table 3:** 

Variable	OR (95 %-KI)	*p*
*Geschlecht*		
Weiblich	1,04 (0,89–1,20)	0,623
Männlich (Ref.)	1	–
*Altersgruppen in Jahren*		
50–64	4,48 (2,99–6,73)	< 0,001
65–75	3,33 (2,22–4,99)	< 0,001
74–89	1,28 (1,07–1,52)	< 0,001
90+ (Ref.)	1	–
*Partnerschaft*		
Ohne Partner*in	1,28 (1,07–1,52)	0,007
Mit Partner*in (Ref.)	1	–
*Veränderung sozialer Unterstützung im Zeitverlauf 2017–2018/2019*		
Seit 2017 ohne soziale Unterstützung	1,36 (1,06–1,75)	0,016
Seit 2017 mit sozialer Unterstützung	0,97 (0,78–1,21)	0,808
In 2018/2019 mit sozialer Unterstützung	0,86 (0,64–1,16)	0,334
In 2018/2019 ohne soziale Unterstützung (Ref.)	1	–
*Veränderung der Anzahl der Diagnosen im Zeitverlauf 2017–2018/2019*		
Seit 2017 mind. 2 Diagnosen	1	–
In 2019 Reduktion zu einer Diagnose	2,49 (2,08–2,97)	< 0,001
*Erstdiagnosen in 2018/2019*		
Chronisch obstruktive Lungenerkrankung (COPD; Ref.)	1	–
Demenz (inkl. Alzheimer)	0,04 (0,01–0,18)	< 0,001
Polyarthrose	0,98 (0,67–1,43)	0,914
Herzinsuffizienz	1,23 (0,83–1,82)	0,294
Rückenschmerzen	2,33 (1,62–3,36)	< 0,001
Hirninfarkt	0,82 (0,51–1,30)	0,392
Sonstige Arthrose	0,93 (0,59–1,46)	0,736
Sonstige psychische Erkrankung	0,31 (0,15–0,63)	0,001
Gonarthrose	2,37 (1,54–3,64)	< 0,001
Chronische ischämische Herzkrankheit	2,08 (1,31–3,31)	0,002
Andere Diagnosen	1,11 (0,85–1,44)	0,440
Nagelkerkes R^2^	0,136	–

*Ref.* Referenzkategorie, *KI* Konfidenzintervall

## Diskussion

Die vorliegende Studie untersuchte soziodemografische und gesundheitsbezogene Determinanten im Hinblick auf die Vermeidung von Pflegebedürftigkeit, wobei soziale Determinanten im besonderen Fokus standen. Erstmalig wurden hierfür Quer- und Längsschnittdaten aus Begutachtungen des MD verwendet. Mit Nutzung dieser Datengrundlage betrat die Studie „Gesundheitsverläufe im Alter: Wege in die Pflegebedürftigkeit“ gleichsam Neuland:

1. Erstmals sollten mit den Angaben aus den Begutachtungsverfahren Datensätze breit und systematisch analysiert werden, die ursprünglich nicht zur Ableitung von Schutz- und Risikofaktoren im Vorfeld einer Pflegebedürftigkeit erhoben worden sind. Im Mittelpunkt der Studie stand aber gerade das Bemühen, aus den Begutachtungen Hinweise zu präventiv oder rehabilitativ nutzbaren Potenzialen am Übergang in eine Pflegebedürftigkeit zu erhalten.
2. Das Forschungsinteresse zielte – neben den diagnostischen Angaben – insbesondere auch auf mögliche *soziale* Einflussfaktoren am Übergang in eine Pflegebedürftigkeit, wie bspw. Unterstützungspotenziale durch Partnerschaften, Verwandte oder andere soziale Netzwerke, wie Freund*innen oder Nachbar*innen.

### Profil von Antragstellenden ohne Feststellung einer Pflegebedürftigkeit

Die dargelegten Analysen der Begutachtungsdaten des MD aus den Jahren 2017 und 2018/2019 fokussierten insbesondere die Gruppe von Antragstellenden, die über den betrachteten Zeitraum hinweg ohne Pflegegradeinstufung blieben. Die Beschreibung dieser Gruppe sollte entsprechend Hinweise zu etwaigen präventiven oder rehabilitativen Potenzialen im Vorfeld einer Pflegebedürftigkeit liefern. Dabei ist zunächst festzuhalten, dass die aus den Begutachtungen 2017 beschriebenen Charakteristika in der Nachverfolgung bis Ende 2019 stabil sind: So blieben Jüngere (unter 75-Jährige), Personen ohne Partnerschaft und Antragstellende, die über keine soziale Unterstützung berichteten, mit höherer Wahrscheinlichkeit ohne Pflegegrad. Zudem zeigten sich die demenziellen Erkrankungen als Schlüsseldiagnose insofern, als dass das Vorliegen einer Diagnose aus diesem Formenkreis mit einem deutlich höheren Risiko für einen Pflegegrad verbunden war. Umgekehrt hatten Antragstellende ohne kognitive und psychische Einschränkungen höhere Chancen, ohne Pflegegrad zu bleiben. Dieser Befund ordnet sich in eine bereits länger bekannte Studienlage ein, die die Demenz als bedeutsamen Risikofaktor für die Entstehung einer Pflegebedürftigkeit beschreibt [4, 5, 11–13].

In den vorliegenden Daten zeigt sich mithin das Profil einer Gruppe alleinlebender, unter 75-Jähriger, vielfach ohne Partnerschaft und weitgehend ohne kognitive oder psychische Einschränkungen, häufiger hingegen mit Erkrankungen aus dem muskuloskelettalen Formenkreis. In dieser hochvulnerablen Population bilden sich Ressourcen- und Risikokonstellationen ab, die im betrachteten Zeitraum in der Summe eine noch so weit selbständige Lebensführung erlaubten, dass kein Pflegegrad zuerkannt wurde. Obgleich das Begutachtungsinstrument des MD zur Feststellung einer Pflegebedürftigkeit nach SGB XI insbesondere im Hinblick auf Daten zu den sozialen Bezügen der Antragstellenden teilweise nur eingeschränkte Informationen lieferte, erlaubt die vorgenommene Profilbildung dennoch einige Ableitungen zu präventiven Potenzialen – bei offenkundigen Limitationen.

### Stärken und Limitationen

Zu den Stärken der vorliegenden Studie gehören die Kombination aus nicht durch selektiven Dropout betroffenen Routinedaten des MD, die ärztliche Diagnosen enthielten, mit sozialen Determinanten, die sonst nur aus Beobachtungsstudien mittels Befragungen gewonnen werden können. Die substanzielle Stichprobengröße im Querschnitt verbunden mit der Möglichkeit einer längsschnittlichen Analyse einer Subkohorte stellt eine weitere Stärke dar. Das Nutzbarmachen von Routinedaten des Gesundheitssystems für die Versorgungsforschung und Public Health stellt hierbei eine weitere methodische Stärke dar, wobei die aktuellen Grenzen dieses Datentyps ebenso ausgelotet wurden.

Mithilfe des Text-Mining-Verfahrens zur Gewinnung von Informationen zu Unterstützungspotenzialen aus Freitextangaben konnten Erkenntnisse zu Prädiktoren der Feststellung von Pflegebedürftigkeit generiert werden. Trotz Validierung der Suchergebnisse [10] kann jedoch nicht gänzlich ausgeschlossen werden, dass in Einzelfällen das Suchergebnis nicht die tatsächlichen Verhältnisse abbildet. Demzufolge können Fälle auftreten, in denen z. B. von einer Partnerschaft berichtet wird, es sich dabei aber um eine/einen verstorbene*n Partner*in handelt. Ebenso ist es denkbar, dass Personen nicht explizit von einer Partnerschaft berichten, aber tatsächlich in einer solchen leben und infolgedessen auf Unterstützung zurückgreifen können. Einige der in die Regressionsanalyse einbezogenen Variablen (z. B. Demenz) weisen zudem nur geringe Fallzahlen auf. Daraus resultieren zum Teil breite Konfidenzintervalle, die auf unsichere Schätzer schließen lassen.

Zu den weiteren Schwächen der Studie gehört der Inferenzschluss (d. h. der Rückschluss von Eigenschaften einer Stichprobe auf Eigenschaften einer Grundgesamtheit) der Frage nach Prävention von Pflegebedürftigkeit innerhalb der spezifisch gewählten Population. Die Gründe älterer Menschen und ihrer Familien, einen Pflegegrad zu beantragen, aber auch die Chance auf Nichtfeststellung von Pflegebedarf lassen eine geradlinige Ableitung von Präventionspotenzial nur eingeschränkt zu. Die komplementären Befunde aus Beobachtungsstudien können hier genutzt werden, um die vorliegenden Ergebnisse einzuordnen und zu kreuzvalidieren. Zukünftige Forschung sollte zudem die Selektivität oder Passung der vorliegenden MD-Daten gegenüber der Grundgesamtheit von älteren Menschen mit und ohne tatsächlichen Pflegebedarf untersuchen.

### Präventive Potenziale im Vorfeld einer Pflegebedürftigkeit

Im Fazit kann die beschriebene Gruppe, die trotz mindestens zweimaliger Begutachtung im Zeitraum 2017 bis Ende 2019 ohne Pflegegrad blieb, als eine Referenzgruppe mit Blick auf Potenziale für den Erhalt einer fragilen funktionalen Gesundheit betrachtet werden, aus der sowohl Hinweise zur krankheitsbezogenen Prävention als auch zur Bedeutung sozialer Konstellationen abzuleiten sind. Krankheitsprävention muss für diese Gruppe vor allem auf die Entwicklung von Strategien zur Vermeidung oder Verzögerung von kognitiven Abbauprozessen fokussieren und dabei bereits bei den unter 75-Jährigen ansetzen. Empfehlungen richten sich aus pathogenetischer und salutogenetischer (d. h. ressourcenorientierter) Perspektive sowohl auf medikamentöse Ansätze – etwa zur Verlangsamung von pathologischen Abbauprozessen – als auch auf nichtmedikamentöse Strategien der Ressourcenstärkung, wie gezieltes kognitives Training, die Ermöglichung von sozialer Teilhabe sowie den Einsatz von technischen Hilfsmitteln (z. B. Orientierungshilfen) zur Kompensierung leichterer kognitiver Einschränkungen. Zum anderen sind angesichts häufiger Diagnosen in der beschriebenen Gruppe (vor allem im muskuloskelettalen Formenkreis) angemessene medikamentöse und nichtmedikamentöse Ansätze zur Schmerztherapie ebenso zu empfehlen wie zielgruppenadäquate, bewegungsfördernde und mobilisierende Angebote [14, 15]. Insgesamt ist tertiärpräventives und rehabilitatives Handeln deutlich zu stärken.

Soziale Risiken für den Eintritt einer Pflegebedürftigkeit können in dieser Gruppe mittelfristig vor allem in vielfach fehlenden engen Beziehungen und entsprechend brüchigen oder nicht vorhandenen Unterstützungspotenzialen gesehen werden. Diese Defizite entfalten in Zuständen hoher Fragilität mit steigendem Alter zunehmende Risiken für den Erhalt von Selbständigkeit. Empfehlungen richten sich aus einer salutogenetischen Perspektive auf die Förderung von inter- und intragenerationalen Wohnformen. Diese sind geeignet, Netzwerke enger und verbindlicher sozialer Beziehungen zu knüpfen. Die hierdurch entstehenden sozialen Ressourcen in Form von Unterstützungspotenzialen und ein erhöhtes Aktivitätslevel [16] können gerade bei frühzeitiger Aktivierung funktionale Gesundheit stärken und zudem helfen, Einsamkeit als gesundheitliches Risiko zu vermeiden. Angesprochen sind hier konkret Akteure auf politischen Entscheidungsebenen (rechtliche Rahmenbedingungen für neue Wohnformen, Steuerung von monetären Ressourcen) sowie Akteure der privaten und kommunalen Bauwirtschaft.

Aus einer sozialversicherungsrechtlichen Perspektive muss die beschriebene Gruppe vulnerabler Personen an der Schwelle zu einer Pflegebedürftigkeit im Zentrum eines konsequent präventiv und rehabilitativ ausgerichteten Leistungsgeschehens stehen [17, 18]. Die oben skizzierten Ansatzpunkte zur krankheitsbezogenen Prävention wie auch zur Stärkung individueller und sozialer Ressourcen sollten als Bestandteil des Begutachtungsverfahrens standardmäßig allen Antragstellenden unterbreitet werden, denen nach Prüfung noch kein Pflegegrad gemäß SGB XI zuerkannt wurde. Ein systematisches präventives und rehabilitatives Handeln vor dem drohenden Übergang in eine Pflegebedürftigkeit könnte demnach einen wichtigen Beitrag leisten – sowohl für die Gesundheit und Lebensqualität der Betroffenen und deren Angehörigen als auch mit Blick auf die Versorgungslücke zwischen Angebot und Nachfrage nach pflegerischen Leistungen.
